# SIRT1 regulates hepatic vldlr levels

**DOI:** 10.1186/s12964-024-01666-y

**Published:** 2024-05-28

**Authors:** Mona Peyman, Anna Babin-Ebell, Rosalía Rodríguez-Rodríguez, Matilde Rigon, David Aguilar-Recarte, Joan Villarroya, Anna Planavila, Francesc Villarroya, Xavier Palomer, Emma Barroso, Manuel Vázquez-Carrera

**Affiliations:** 1 Department of Pharmacology, Toxicology and Therapeutic Chemistry, Faculty of Pharmacy and Food Sciences, Barcelona, Spain; 2 Institute of Biomedicine of the University of Barcelona (IBUB), University of Barcelona, Barcelona, 08028 Spain; 3 Spanish Biomedical Research Center in Diabetes and Associated Metabolic Diseases (CIBERDEM)-Instituto de Salud Carlos III, Madrid, 28029 Spain; 4 Pediatric Research Institute-Hospital Sant Joan de Déu Esplugues de Llobregat, Barcelona, 08950 Spain; 5 Basic Sciences Department, Faculty of Medicine and Health Sciences, Universitat Internacional de Catalunya (UIC), Sant Cugat del Vallès, Barcelona, 08017 Spain; 6 Spanish Biomedical Research Center in Physiopathology of Obesity and Nutrition (CIBEROBN)-Instituto de Salud Carlos III, Madrid, 28029 Spain; 7 Department of Biochemistry and Molecular Biomedicine, Faculty of Biology, University of Barcelona, Barcelona, 08028 Spain

**Keywords:** MASLD, SIRT1, VLDLR, HIF-1α, ER stress

## Abstract

**Background:**

Endoplasmic reticulum (ER) stress-mediated increases in the hepatic levels of the very low-density lipoprotein (VLDL) receptor (VLDLR) promote hepatic steatosis by increasing the delivery of triglyceride-rich lipoproteins to the liver. Here, we examined whether the NAD(^+^)-dependent deacetylase sirtuin 1 (SIRT1) regulates hepatic lipid accumulation by modulating VLDLR levels and the subsequent uptake of triglyceride-rich lipoproteins.

**Methods:**

Rats fed with fructose in drinking water, *Sirt1*^−/−^ mice, mice treated with the ER stressor tunicamycin with or without a SIRT1 activator, and human Huh-7 hepatoma cells transfected with siRNA or exposed to tunicamycin or different inhibitors were used.

**Results:**

Hepatic SIRT1 protein levels were reduced, while those of VLDLR were upregulated in the rat model of metabolic dysfunction-associated steatotic liver disease (MASLD) induced by fructose-drinking water. Moreover, *Sirt1*^−/−^ mice displayed increased hepatic VLDLR levels that were not associated with ER stress, but were accompanied by an increased expression of hypoxia-inducible factor 1α (HIF-1α)-target genes. The pharmacological inhibition or gene knockdown of SIRT1 upregulated VLDLR protein levels in the human Huh-7 hepatoma cell line, with this increase abolished by the pharmacological inhibition of HIF-1α. Finally, SIRT1 activation prevented the increase in hepatic VLDLR protein levels in mice treated with the ER stressor tunicamycin.

**Conclusions:**

Overall, these findings suggest that SIRT1 attenuates fatty liver development by modulating hepatic VLDLR levels.

**Supplementary Information:**

The online version contains supplementary material available at 10.1186/s12964-024-01666-y.

## Background

The first stage in the development of metabolic dysfunction-associated steatotic liver disease (MASLD, formerly referred to as nonalcoholic fatty liver disease [NAFLD]) is liver steatosis, which is defined as a condition where excessive levels of triglycerides accumulate in the liver (at least 5% of liver weight) [1]. Hepatic triglyceride levels are regulated by multiple mechanisms such as de novo synthesis, fatty acid oxidation, lipolysis, dietary fat consumption, and the hepatic secretion of lipoprotein particles [2]. Not much is known about the role played by the uptake of lipoproteins, such as very low-density lipoproteins (VLDL) and chylomicrons that predominantly transport triglycerides in the plasma, in the development of hepatic steatosis. Interestingly, it has been reported that the endoplasmic reticulum (ER) stress-mediated increase in the levels of the VLDL receptor (VLDLR) results in remarkable hepatic steatosis via enhanced triglyceride-rich lipoprotein delivery to the liver [3]. VLDLR is widely expressed in the brain, heart, skeletal muscle and adipose tissue, whereas its expression is very low in the liver under normal conditions [4, 5]. VLDLR belongs to the low-density lipoprotein (LDL) receptor family. It binds apolipoprotein E (apoE) and triglyceride-rich lipoproteins such as chylomicrons and VLDL, promoting lipid entry into the cell through receptor-mediated endocytosis or by lipoprotein lipase-dependent lipolysis [6–9]. As a result of this function, a link has been established between VLDLR content and plasma triglyceride levels [10].

VLDLR expression has been reported to be upregulated by several transcription factors, including activating transcription factor 4 (ATF4) in the liver during ER stress [3], peroxisome proliferator-activated receptor (PPAR) γ in adipose tissue [11] and hypoxia-inducible factor 1α (HIF-1α) in the heart [12], contributing to lipid deposition or supply in all these tissues. In addition, the increase in hepatic VLDLR levels caused by fenofibrate through PPARα activation plays an essential role in the triglyceride-lowering effect of this drug [13].

Sirtuin 1 (SIRT1) is a NAD(^+^)-dependent deacetylase, and a key regulator of MASLD through the regulation of lipid metabolism, oxidative stress and inflammation in the liver [14]. In fact, SIRT1 overexpression reduces the level of oxygen consumption in MASLD and relieves oxidative stress [15]. In addition, the selective SIRT1 activator SRT1720 attenuates high-fat diet (HFD)-induced liver steatosis [16]. Likewise, SIRT1 activation deacetylates the p65 subunit of NF-κB at lysine 310 and inhibits this inflammatory transcription factor [17]. Consistent with these actions, heterozygous SIRT1 knockout (*Sirt1*^+/−^) mice fed an HFD exhibit hepatic steatosis with significant increases in lipid content and liver inflammation [18]. However, it is currently unknown if SIRT1 regulates hepatic lipid accumulation by modulating the levels of VLDLR and the subsequent uptake of triglyceride-rich lipoproteins. Here, we show that the presence of hepatic steatosis in a model of MASLD induced by fructose-drinking water was accompanied by a reduction in hepatic SIRT1 protein levels and an upregulation of VLDLR levels, suggesting a potential relationship between these two proteins. Interestingly, *Sirt1*^−/−^ mice exhibited an increase in hepatic VLDLR levels that was not associated with ER stress, but was accompanied by an increase in the expression of HIF-1α-target genes. The pharmacological inhibition or gene knockdown of SIRT1 increased VLDLR protein levels in a human hepatic cell line and this increase was abolished by the pharmacological inhibition of HIF-1α. Finally, SIRT1 activation in mice prevented the increase in hepatic VLDLR protein levels caused by ER stress. Collectively, the findings of this study indicate that the protective effect of SIRT1 in MASLD includes the modulation of hepatic VLDLR levels.

## Methods

### Reagents

Control siRNA and SIRT1 siRNA were purchased from Santa Cruz (Dallas, TX, USA). PX-478 and SRT1720 were purchased from Apexbio (Houston, TX, USA), tunicamycin from Tocris (Bristol, UK) and EX-527 from RayBiotech (Peachtree Corners, GA, USA). Plasma triglyceride levels were analyzed using a commercial kit (Spinreact SA, St. Esteve d’en Bas, Spain).

### Animal treatment

Three-month-old male Sprague-Dawley rats (Envigo, Barcelona, Spain) were housed under conditions of constant humidity (40–60%) and temperature (20–24 °C), with a light/dark cycle of 12 h. Rats were randomly assigned to two groups: control (CT) and fructose (*n* = 5 in each). In addition to normal chow, the rats had free access to a 10% (w/v)-fructose solution or plain tap water for 3 weeks. In the fructose group, one rat was euthanized before the end of the experimental period, due to a growing tumor. Thus, the final n for the fructose group was 4.

For the glucose tolerance test (GTT), animals received 2 g/kg body weight of glucose via an intraperitoneal injection and blood was collected from the tail vein after 0, 15, 30, 60 and 120 min.

Livers from male *Sirt1* knockout (*Sirt1*^−/−^) mice (4-week-old; 129Sv: B6) and their wild-type littermates (*Sirt1*^+/+^) were used [19]. To confirm the genotype of the mice, a PCR analysis was conducted on extracted tail DNA using oligonucleotides (forward: 5’-CTTGCACTTCAAGGGACCAAGT-3’. Reverse: 5’-CGTCACTAACCATGACACTGAAGG-3’ and 5’-TCTGGCCAAAGTAGGCAGACA-3’), generating, respectively, a Sirt1 endogenous amplicon of 370 bp and a knockout amplicon of 200 bp.

Three-month-old male mice fed standard chow were randomly assigned to three groups: CT, tunicamycin, and tunicamycin + SRT1720 (*n* = 4 in each). The mice received one daily p.o. dose of 200 mg/kg/day of the SIRT1 activator SRT1720 [20] or carboxymethylcellulose (CMC) (volume administered, 1 mL/kg) as vehicle for 5 days. Twenty-four hours before the sacrifice, the mice were administered an i.p. injection of DMSO (vehicle, control, CT) or tunicamycin (1 mg/kg body weight). At the end of the treatment, the mice were sacrificed, and the obtained serum and liver samples were frozen in liquid nitrogen and then stored at -80ºC.

Animal experimentation complied with the Guide for the Care and Use of Laboratory Animals published by the US National Institutes of Health (8th Edition: National Academies Press; 2011). All procedures were approved by the Bioethics Committee of the University of Barcelona, as stated in Law 5/21 July 1995 passed by the Generalitat de Catalunya. The animals were treated humanely, and all efforts were made to minimize both animal numbers and suffering.

### Liver triglyceride content

Liver triglycerides were extracted according to the method of Bligh and Dyer [21]. The lipid extract was evaporated under a stream of nitrogen gas, redissolved in absolute ethanol and quantified using a commercial kit (Spinreact SA).

### Liver histology

For histological staining studies, samples were fixed in formalin, paraffin embedded and 4 μm sections were obtained. Oil Red O staining (Sigma Aldrich) was performed on frozen 10-µm liver sections. Fifteen images at 200x magnification were captured to quantify lipid droplets, which were evaluated as the red-stained area per total area with ImageJ.

### Cell culture

Human Huh-7 hepatoma cells (kindly donated by Dr. Mayka Sanchez from the Josep Carreras Leukemia Research Institute, Barcelona, Spain) were cultured in DMEM supplemented with 10% fetal bovine serum and 1% penicillin-streptomycin at 37 °C under 5% CO_2_.

Huh-7 cells were transiently transfected with 100 nM siRNA against SIRT1 or siRNA control (Santa Cruz) in Opti-MEM medium (Thermo Fisher, MA, USA), using Lipofectamine 2000 (Invitrogen, Carlsbad, CA, USA) according to the manufacturer’s instructions. Different compounds were tested after 24 h of transfection.

Huh-7 cells were exposed to 10 µM EX-527, 20 µM PX-478, 10 µM tunicamycin or 10 mM SRT1720 [22] for 24 h.

### VLDL uptake assay

VLDLs labeled with 1,1′-dioctadecyl-3,3,3′,3’-tetramethylindocarbocyanine perchlorate (DiI) were obtained from Alfa Aesar (cat. no. J65568). Huh-7 cells were pretreated in serum-free media with 10 µM EX-527 or with this compound plus 20 µM PX-478 for 24 h prior to a 1-h incubation with 10 µg/ml of DiI-VLDL. Surface-bound DiI-VLDL was removed with acid-wash buffer (0.5 M acetic acid with 150 mM NaCl, pH 2.5). Cells were washed with DPBS containing calcium and magnesium, lysed in 1% SDS and 0.1 M NaOH, transferred to a black 96-well half-area plate (Greiner Bio-One), and assessed using a Varioskan microplate reader (excitation/emission: 520/580 nm; Molecular Devices). Fluorescence was corrected for protein amount.

### Quantitative RT-PCR

The relative levels of specific mRNAs were assessed by real-time RT-PCR, as previously described [23]. Values were normalized to the expression levels of glyceraldehyde 3-phosphate dehydrogenase (*Gapdh*) or adenine phosphoribosyltransferase (*Aprt*), and measurements were performed in triplicate. All expression changes were normalized to that of the untreated control. The primer sequences used for real-time RT-PCR are shown in Supplementary Table 1.

### Immunoblotting

The isolation of total protein extracts was performed as described elsewhere [23]. Proteins (30 µg) were separated by SDS-PAGE on 8–12% acrylamide gels and transferred onto Immobilon polyvinylidene difluoride membranes (Millipore). Incubation with the primary antibody was performed overnight in a cold room in the WestVision™ Block and Diluent solution (cat. no.: SP-7000, Vector Labs, CA, USA). The membranes were washed five times with a TBS-0.1% Tween solution and incubated with a horseradish peroxidase-conjugated secondary antibody (GE Healthcare) in PBS-0.1% Tween containing 3% BSA for one hour at room temperature. After incubation with the secondary antibody, the membranes were washed three times with a PBS-0.1% Tween solution and incubated with the detection reagent. Protein bands were detected with the Western Lightning® Plus-ECL chemiluminescence reagent kit (PerkinElmer, Waltham, MA, USA). The size of the detected proteins was estimated using protein molecular mass standards (Bio-Rad, Barcelona, Spain). Signal acquisition was performed using the Bio-Rad ChemiDoc apparatus and quantification of the immunoblot signal was performed with the Bio-Rad Image Lab software. The results for protein quantification were normalized to the levels of a control protein to avoid unwanted sources of variation. Immunoblotting was performed with antibodies against ATF4 (#1185, Cell Signaling Technology, Danvers, MA, USA), β-actin (A5441, Sigma), BiP/78-kDa glucose-regulated protein (GRP78) (#3183, Cell Signalling Technology), CHOP (GTX112827, Genetex, Irvine, CA, USA), FGF21 (sc-22,920, Santa Cruz Biotechnology Inc.), HIF-1α (sc-10,790, Santa Cruz Biotechnology Inc.), NQO1 (sc-393,736, Santa Cruz Biotechnology Inc.), Ac-p53 (#2525, Cell Signaling Technology), SIRT1 (ab189494, Abcam), TRB3 (sc-365,842, Santa Cruz Biotechnology Inc.), tubulin (T6074, Sigma) or VLDLR (AF2258, R&D Systems).

### Statistical analysis

Results are expressed as the mean ± SEM. Significant differences were assessed by either Student’s t-test or one-way ANOVA, according to the number of groups compared, using the GraphPad Prism program (version 9.0.2) (GraphPad Software Inc., San Diego, CA, USA). When significant variations were found by ANOVA, Tukey’s post-hoc test for multiple comparisons was performed only if F achieved a p value < 0.05. Differences were considered significant at *p* < 0.05.

## Results

### VLDLR levels are increased and SIRT1 protein levels are reduced in the livers of rats supplemented with liquid fructose

**Fig. 1:**
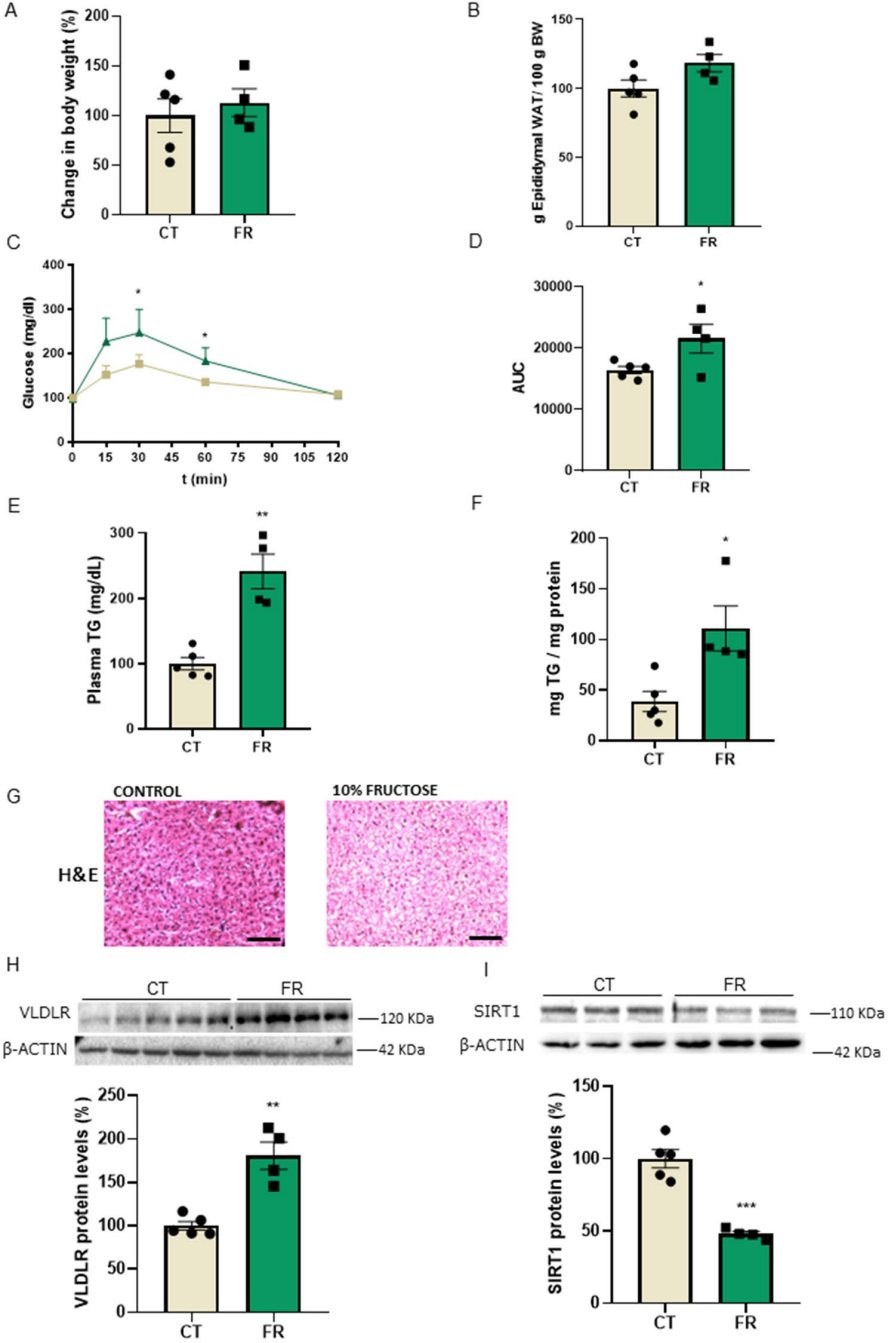
Hepatic steatosis induced by liquid fructose in rats results in an increase in VLDLR levels and a reduction in SIRT1 protein levels. (**A**) Changes in body weight in rats with free access to plain tap water (control, CT) or to a 10% (w/v)-fructose (FR) solution for 3 weeks. (**B**) Epididymal adipose tissue. (**C**) Glucose tolerance test (GTT) and (**D**) area under the curve (AUC) in CT and FR rats. (**E**) Plasma triglyceride (TG) levels. (**F**) Hepatic TG levels. (**G**) Representative images of liver sections with hematoxylin–eosin (H&E) staining in CT and FR rats. Scale bar: 100 μm. Immunoblot analysis of (**H**) VLDLR and (**I**) SIRT1 in the livers of CT and FR rats. Data are presented as the mean ± SEM. Significant differences were established by Student’s t-test. *p < 0.05 and **p < 0.01 vs. CT. *n* = 4 or 5 per group

First, we examined the protein levels of VLDLR in the livers of rats supplemented with fructose, a well-known inducer of fatty liver [24]. Supplementation with 10% liquid fructose for 21 days did not affect either body weight (Fig. 1A) or the epididymal fat depot (Fig. 1B), but it resulted in glucose intolerance, as demonstrated by the GTT (Fig. 1C, D). In addition, fructose ingestion increased plasma triglyceride levels (Fig. 1E) and caused hepatic steatosis, as revelaed by the quantification of triglyceride accumulation in the liver (Fig. 1F) and hematoxylin & eosin (H&E) staining (Fig. 1G). Interestingly, even the induction of a mild liver steatosis with a low percentage of fructose for a relatively short period of time resulted in an increase in the protein levels of VLDLR (Fig. 1H), while the protein levels of SIRT1 were reduced (Fig. 1I).

### *Sirt1*^*−/−*^ mice show increased hepatic protein levels of VLDLR in the absence of ER stress

**Fig. 2:**
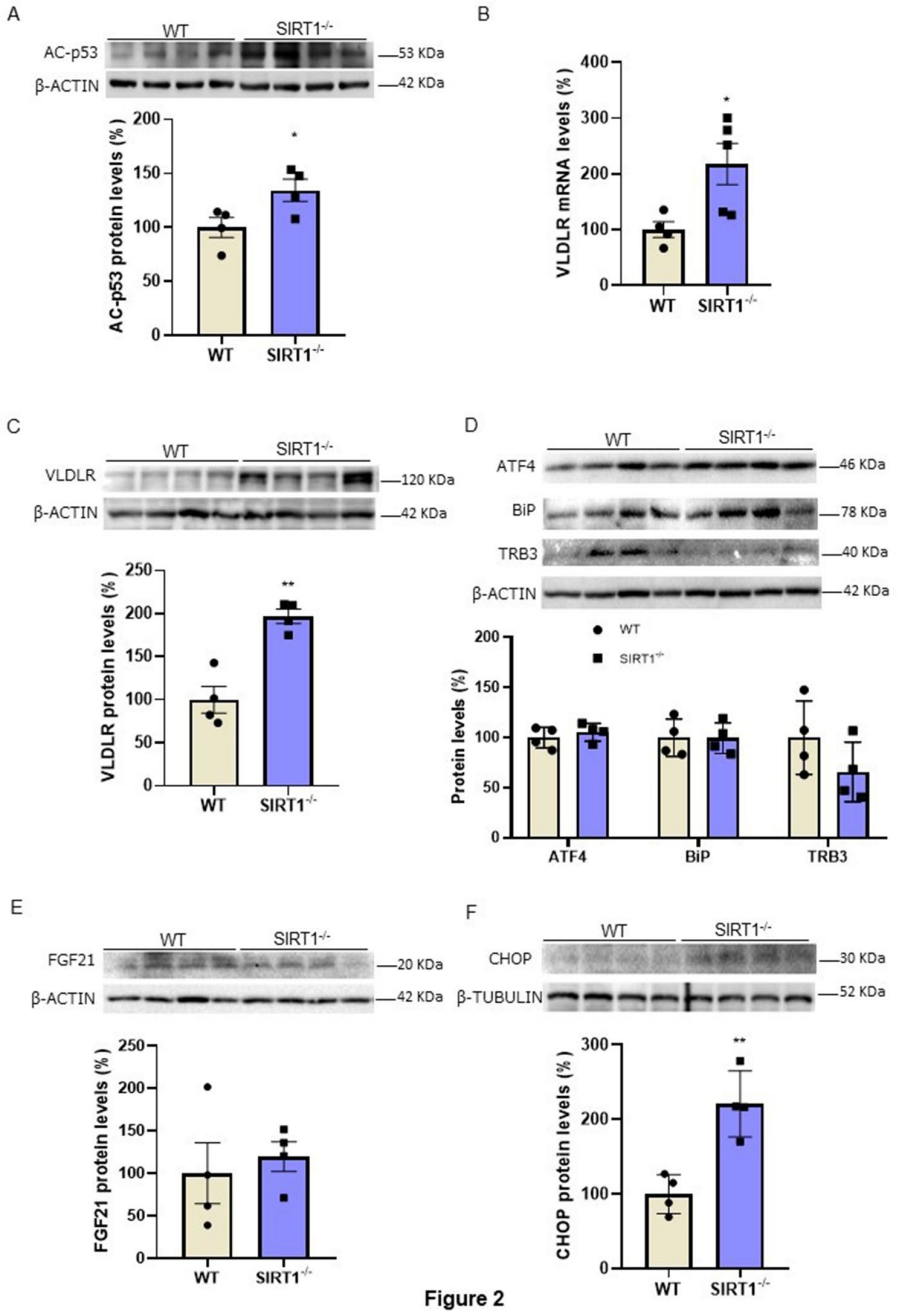
VLDLR levels are increased in the livers of*Sirt1*^*−/−*^mice. (**A**) Immunoblot analysis of acetylated (Ac)-p53 in the livers of WT and *Sirt1*^*−/−*^ mice. (**B**) mRNA levels of *Vldlr*. Immunoblot analysis of (**C**) VLDLR, (**D**) ATF4, BiP/GRP78, TRB3, (**E**) FGF21 and (**F**) CHOP in the livers of WT and *Sirt1*^*−/−*^ mice. Data are presented as the mean ± SEM. Significant differences were established by Student’s t-test. *p < 0.05 and **p < 0.01 vs. CT. *n* = 4 or 5 per group

To demonstrate that SIRT1 regulates VLDLR levels, we used the livers of wild-type and *Sirt1*^−/−^ mice (genotyping is shown Supplementary Fig. 1A) that survived to adulthood, since only 24% of *Sirt1*^−/−^ pups survive the first week of life [19]. In the livers of these mice, we assessed the protein levels of acetylated p53, a target of SIRT1 [25]. As expected, *Sirt1* deficiency resulted in increased levels of acetylated p53 (Fig. 2A). Remarkably, the mRNA (Fig. 2B) and protein levels (Fig. 2C) of VLDLR were increased in the livers of *Sirt1*^−/−^ mice compared to wild-type mice, indicating that the absence of this deacetylase might increase VLDLR levels through a transcriptional mechanism. Since VLDLR expression has been reported to be upregulated by the transcription factor ATF4 in the liver during ER stress [3], we determined the levels of ATF4 as well as of other markers of ER stress. No changes were observed in the protein levels of ATF4 in the livers of *Sirt1*^*−/−*^ mice (Fig. 2D). Likewise, no changes were detected in the protein levels of the ER stress markers BiP/GRP78, tribbles 3 (TRB3) (Fig. 2D) and FGF21 (Fig. 2E), with FGF21 reported to be upregulated by ATF4 [26]. However, the protein levels of CHOP were upregulated in the livers of *Sirt1*^*−/−*^ mice (Fig. 2F). The increase in CHOP levels might be related not to the presence of ER stress, but to the upregulation of VLDLR, since a previous study has reported that the absence of VLDLR in white adipose tissue is accompanied by a reduction in CHOP levels [27], suggesting that VLDLR regulates CHOP levels.

### SIRT1 inhibition leads to the upregulation of VLDLR in hepatic cells

**Fig. 3:**
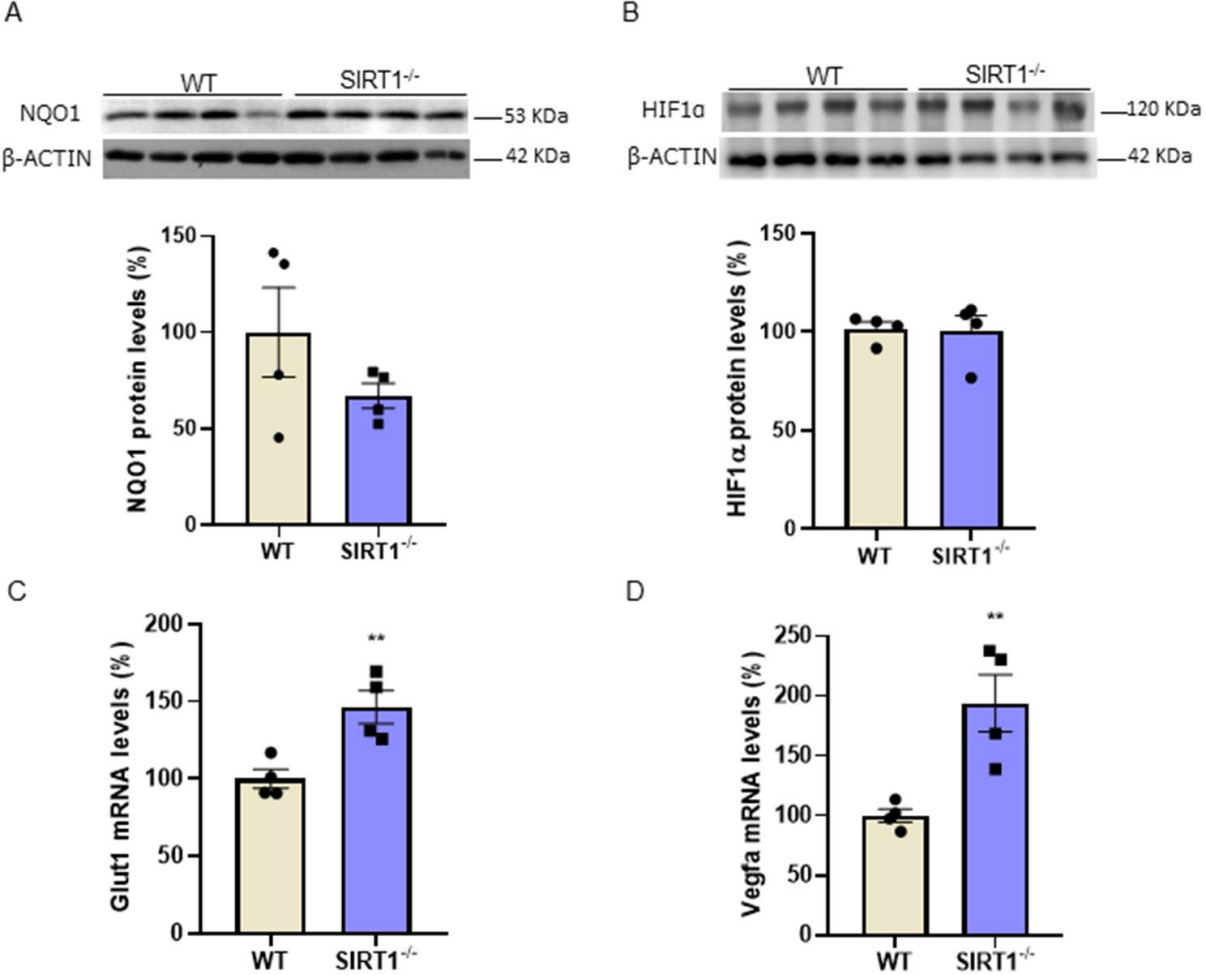
The expression of HIF-1α-target genes is increased in the livers of*Sirt1*^*−/−*^mice. Immunoblot analysis of (**A**) NQO1 and (**B**) HIF-1α in the livers of WT and *Sirt1*^*−/−*^ mice. mRNA levels of (**C**) *Glut1* and (**D**) *Vegfa* in the livers of WT and *Sirt1*^*−/−*^ mice. Data are presented as the mean ± SEM. Significant differences were established by Student’s t-test. *p < 0.05 and **p < 0.01 vs. CT. *n* = 4 per group

**Fig. 4:**
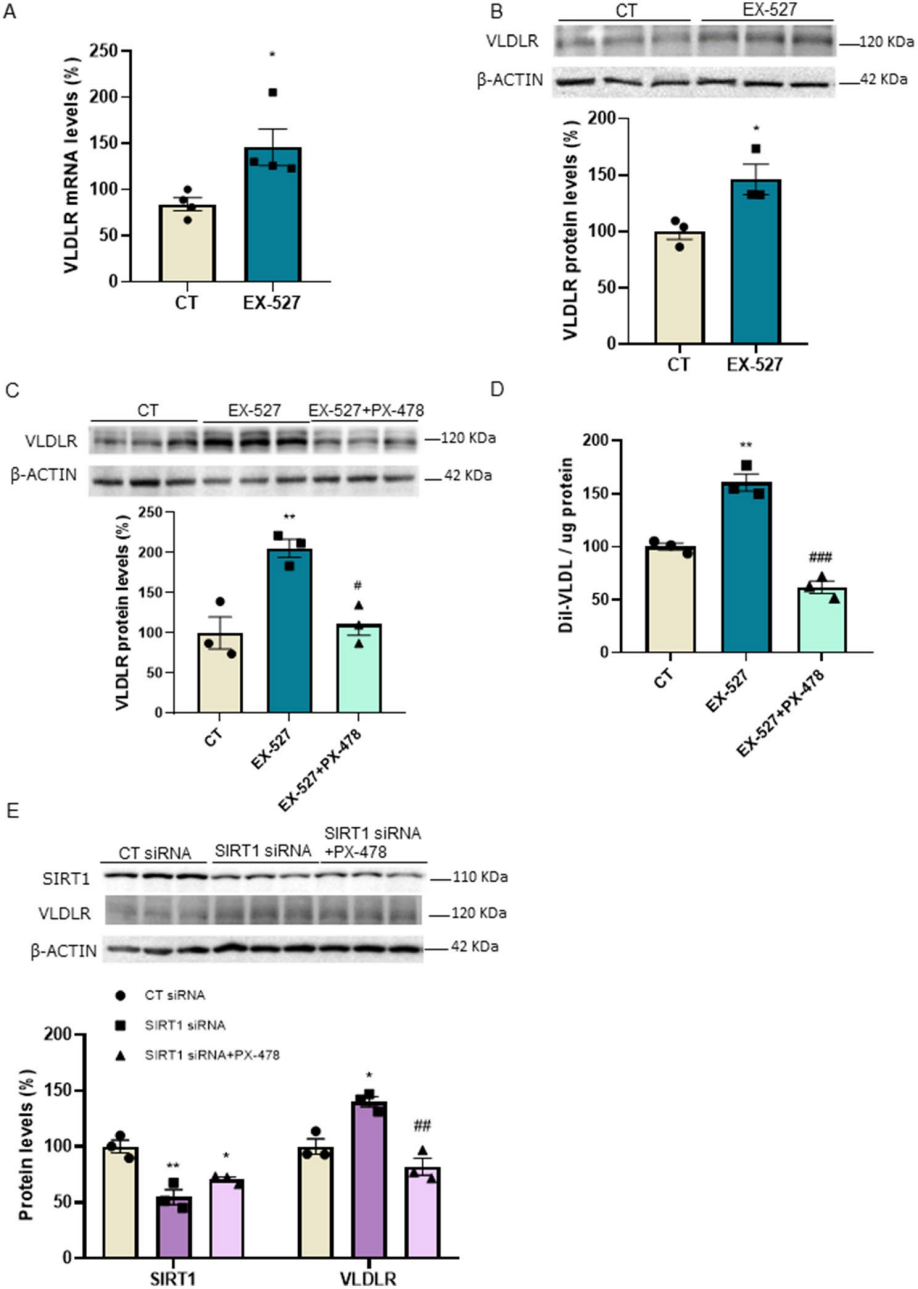
SIRT1 inhibition increases VLDLR levels and VLDL uptake in human Huh-7 cells. (**A**) mRNA and (**B**) immunoblot analysis of VLDLR in human Huh-7 cells in the absence (control, CT) or presence of 10 µM EX-527 for 24 h. Immunoblot analysis of (**C**) VLDLR and (**D**) VLDL uptake in human Huh-7 cells in the absence (control, CT) or presence of 10 µM EX-527, or in the presence of both 10 µM EX-527 and 20 µM PX-478 for 24 h. (**E**) Immunoblot analysis of SIRT1 and VLDLR in Huh-7 cells transfected with control siRNA or SIRT1 siRNA in the absence or presence of 20 µM PX-478. Data are presented as the mean ± SEM. Significant differences were established by Student’s t-test or one-way ANOVA with Tukey’s post-hoc test. *p < 0.05 and **p < 0.01 vs. CT. ^#^*p* < 0.05, ^##^*p* < 0.01, and ^###^*p* < 0.001 vs. EX-527 or SIRT1 siRNA. *n* = 3 or 4 per group

Since VLDLR has been reported to be under the transcriptional control of nuclear factor (erythroid-derived 2)-like 2 (Nrf2) [28], we also examined the protein levels of its target gene NAD(P)H quinone dehydrogenase 1 (NQO1). No changes were observed in the NQO1 protein levels in the livers of *Sirt1*^−/−^ mice (Fig. 3A), making the contribution of Nrf2 to the increase in VLDLR levels unlikely in these mice. Another transcription factor regulating VLDLR expression is HIF-1α [12]. Although no changes were observed in HIF-1α protein levels in the livers of *Sirt1*^−/−^ mice compared to wild-type mice (Fig. 3B), its transcriptional activity might be upregulated, since the hepatic expression of its target genes, *Glut1* (Fig. 3C) and *Vegfa* (Fig. 3D), was increased in the *Sirt1*^−/−^ mice. These findings might suggest that the increased transcriptional activity of HIF-1α might also contribute to elevated VLDLR levels in the livers of *Sirt1*^−/−^ mice. To demonstrate that reduced SIRT1 activity leads to VLDLR upregulation through HIF-1α, we used both a pharmacological and a genetic approach in the human Huh-7 hepatoma cell line. First, we used a potent and selective SIRT1 inhibitor, EX-527 [29, 30]. EX-527 increased the expression of the HIF-1α-target gene *Vegfa* (Supplementary Fig. 1B). Moreover, exposure of Huh-7 cells to EX-527 increased both the expression (Fig. 4A) and the protein levels (Fig. 4B) of VLDLR, supporting the findings obtained in the *Sirt*^*−/−*^ mice. Interestingly, co-incubation of the cells with EX-527 and the HIF-1α inhibitor PX-478 [31] abrogated the increase in VLDLR protein levels caused by EX-527 (Fig. 4C). Next, we assessed whether the changes in VLDLR levels affected the uptake of VLDL. Consistent with the increase in VLDLR levels caused by EX-527, this compound upregulated VLDL uptake, with the effect of EX-527 blunted in the presence of PX-478 (Fig. 4D).

To further demonstrate that SIRT1 downregulation increases VLDLR levels, we knocked down *SIRT1* expression by transfecting cells with siRNA targeting the *SIRT1* gene. Knockdown of *SIRT1* reduced its protein levels and increased those of HIF-1α (Supplementary Fig. 1C) and VLDLR (Fig. 4E). However, incubation with PX-478 completely abolished the increase in VLDLR levels (Fig. 4E). Collectively, these findings indicate that the reduction in the activity or in the levels of SIRT1 in human Huh-7 hepatic cells results in the upregulation of VLDLR.

### SIRT1 activation ameliorates fatty liver and abolishes the increase in hepatic VLDLR levels caused by ER stress

**Fig. 5:**
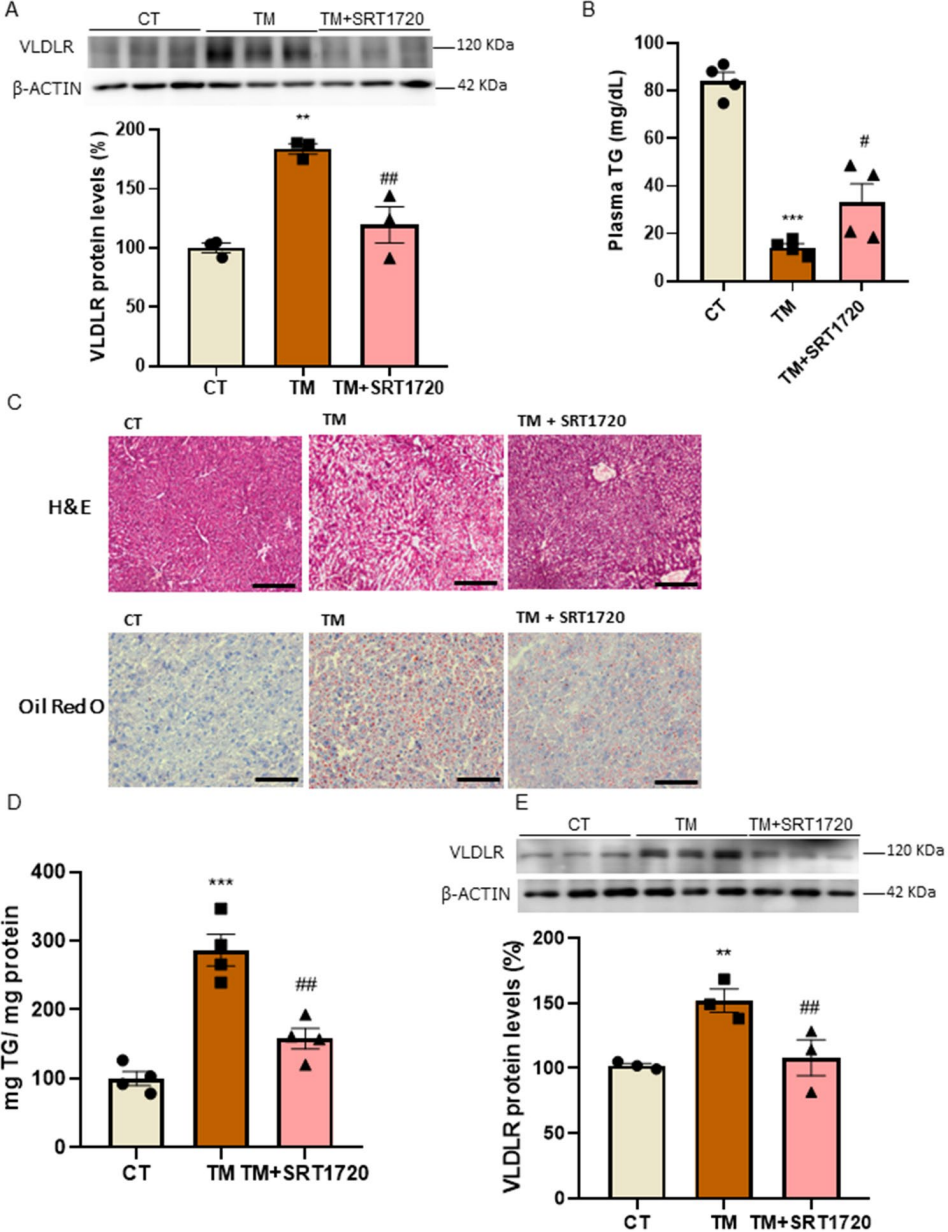
SIRT1 activation prevents the increase in VLDLR levels caused by the ER stressor tunicamycin. (**A**) Immunoblot analysis of VLDLR in human Huh-7 cells in the absence (control, CT) or presence of tunicamycin or in the presence of tunicamycin plus SRT1720 for 24 h (*n* = 3). (**B**) Plasma triglyceride (TG) levels in mice treated with the SIRT1 activator SRT1720 for 5 days and vehicle or tunicamycin for the last 24 h (*n* = 4 animals). (**C**) Representative images of liver sections with hematoxylin-eosin (H&E) and Oil Red O (ORO) staining. Scale bar: 100 μm. (**D**) Hepatic TG levels. (**E**) Immunoblot analysis of VLDLR in the livers of mice. Data are presented as the mean ± SEM. Significant differences were established by one-way ANOVA with Tukey’s post-hoc test. **p < 0.01 and ***p < 0.001 vs. CT. ^#^*p* < 0.05 and ^##^*p* < 0.01 vs. tunicamycin

Since hepatic VLDLR levels are elevated in response to ER stress and as they contribute to ER stress-dependent hepatic steatosis [3], we next evaluated whether SIRT1 activation attenuated VLDLR upregulation and the hepatic steatosis caused by the ER stressor tunicamycin. First, we treated Huh-7 cells with tunicamycin in the presence or absence of the SIRT1 activator SRT1720 [32]. As expected, tunicamycin increased the protein levels of VLDLR, but this increase was completely prevented in the cells co-incubated with SRT1720 (Fig. 5A). We then treated the mice with tunicamycin and with either vehicle or SRT1720. Tunicamycin treatment resulted in a decrease in serum triglyceride levels, which is likely to be the result of the higher uptake of circulating triglyceride-rich lipoproteins by VLDLR, while SRT1720 attenuated this reduction (Fig. 5B). This suggested that the uptake of VLDLs by VLDLR was attenuated. Tunicamycin also led to a clear increase in hepatic triglyceride accumulation, as demonstrated by the H&E and ORO staining (Fig. 5C) and the quantification of this neutral lipid (Fig. 5D). However, treatment with SRT1720 strongly alleviated fatty liver. Consistent with a higher uptake of circulating triglyceride-rich lipoproteins, VLDLR protein levels were increased in the mice treated with tunicamycin (Fig. 5E), whereas the SIRT1 activator abolished this increase. Overall, these findings indicate that SIRT1 activation contributes to the prevention of ER stress-induced fatty liver by VLDLR levels and modulating the serum and hepatic levels of triglycerides.

## Discussion

Liver steatosis is the hallmark of MASLD. The lipid droplets accumulated in the liver mainly consist of triglycerides. Several mechanisms contribute to lipid deposition in the liver, including changes in *de novo* synthesis, fatty acid oxidation, lipolysis, dietary fat consumption, and the hepatic secretion of lipoprotein particles [2]. The contribution of another mechanism, the uptake of triglyceride-rich lipoproteins by the VLDLR, has been precluded by the low expression levels of this receptor in healthy livers [4, 5]. However, the discovery that ER stress stimulates hepatic steatosis by increasing the expression of hepatic VLDLR [3] demonstrates that increased lipoprotein delivery to the liver is a new determinant in hepatic steatosis. The role of VLDLR in the liver is consistent with previous studies reporting that increased VLDLR levels stimulate lipid accumulation in cardiomyocytes [12] and adipocytes [33]. In this study, we show that hepatic SIRT1 downregulation by fructose supplementation is associated with increased VLDLR levels. These findings are consistent with previous studies reporting that one of the mechanisms by which fructose might promote hepatic steatosis is by reducing SIRT1 levels [34, 35]. Moreover, SIRT1 levels have been reported to be reduced in liver biopsies from patients with MASLD [36]. Likewise, mice with a liver-specific knockout of *Sirt1* are prone to hepatic steatosis, while SIRT1 overexpression attenuates hepatic steatosis in mice fed an HFD [37]. The mechanisms by which SIRT1 ameliorates hepatic steatosis include: deacetylation of PPARγ co-activator 1 α (PGC-1α) [38], which enhances the activity of this transcriptional co-activator, thereby leading to increased PPARα activation and the upregulation of genes encoding the enzymes participating in fatty acid oxidation; AMPK activation via the deacetylation and activation of the LKB1 kinase [39, 40]; and the attenuation of lipogenesis by the inhibition of sterol regulatory element-binding protein-1c (SREBP-1c) [41]. Here, we report that SIRT1 regulates VLDLR levels and that this mechanism might modulate the development of fatty liver. Consistent with this, our findings show that the livers of *Sirt1*^−/−^ mice display elevated VLDLR levels. The increased levels of VLDLR in the liver, which is expressed at very low levels in this organ under healthy conditions, may result in an increased lipoprotein delivery to the liver, thereby promoting the accumulation of hepatic triglycerides. Using pharmacological and genetic approaches, we have shown that the reduction in the activity or the levels of SIRT1 results in increased VLDLR levels. Moreover, the upregulation of VLDLR caused by SIRT1 inhibition in hepatic cells results in increased VLDL uptake, with this increase abolished by a HIF-1α inhibitor. A previous study has demonstrated that SIRT1 directly deacetylates HIF-1α, thus inactivating this transcription factor [42].

Several factors (including hyperlipidemia, inflammation, viruses and drugs) have been reported to perturb hepatocyte ER homeostasis in humans, contributing to the dysregulation of hepatic lipid metabolism and liver disease [43]. Severe ER stress may contribute to the development of hepatic steatosis by promoting *de novo* lipogenesis and lipolysis, reducing fatty acid oxidation and disturbing VLDL secretion [43]. In addition, VLDLR upregulation by ER stress increases lipoprotein delivery to the liver, exacerbating fatty liver and reducing serum triglyceride levels as a result of lipoprotein delivery to the liver [3]. In line with this, in our conditions, tunicamycin treatment led to the accumulation of hepatic triglyceride that was accompanied by a reduction in the serum levels of this lipid. Of note, SIRT1 activation in mice treated with the ER stressor tunicamycin prevented the increase in hepatic VLDLR levels and significantly attenuated hepatic steatosis. This is likely to be the result of a reduction in VLDLR uptake, leading to the partial recovery of serum triglyceride levels. In fact, tunicamycin leads to hepatic steatosis by several mechanisms, including an increase in the hepatic levels of VLDLR and the subsequent uptake of VLDL particles [3]. SRT1720, by reducing VLDLR hepatic levels, modulates the uptake of VLDL particles by the liver, resulting in the partial restoration of serum triglyceride levels.

## Conclusions

Altogether, the findings of this study highlight a new regulatory mechanism by which SIRT1 regulates VLDLR levels. During the development of fatty liver, several stimuli such as fructose consumption reduce hepatic SIRT1 levels, exacerbating this condition by increasing VLDLR levels and the subsequent delivery of triglyceride-rich lipoproteins to the liver. In addition, SIRT1 activation can contribute to the improvement of fatty liver by reducing the increase in VLDLR levels caused by ER stress during MASLD.

.

### Electronic supplementary material

Below is the link to the electronic supplementary material.

Supplementary Material 1

Supplementary Material 2

Supplementary Material 3

Supplementary Material 4

## Data Availability

The source data for this study are available as a Source Data file or from the corresponding author upon reasonable request.
