# Supplementary material for: SIRT1 regulates hepatic vldlr levels
Source: Cell Commun Signal. 2024 May 28;22:297. doi: 10.1186/s12964-024-01666-y (PMC11134955; doi:10.1186/s12964-024-01666-y)
Supplement: Supplementary file 1 — Supplementary Material 1 [file 12964_2024_1666_MOESM1_ESM.docx]

**Supplementary Table 1**

| Primer sequences |  |  |
| --- | --- | --- |
|  | Forward | Reverse |
| *VLDLR* | TCCAATGGCCTAATGGAAT | AGCATGTGCAACTTGGAAT |
| *Glut1* | GCCCCCAGAAGGTTATTGA | CGTGGTGAGTGTGGTGGAT |
| *Vegfa* | GCTGTAACGATGAAGCCCTG | GAGAGGTCTGGTTCCCGAAA |
| *Gapdh* | TGTGTCCGTCGTGGATCTGA | CCTGCTTCACCACCTTCTTGA |
