# Supplementary figures and images for: SIRT1 regulates hepatic vldlr levels
Source: Cell Commun Signal. 2024 May 28;22:297. doi: 10.1186/s12964-024-01666-y (PMC11134955; doi:10.1186/s12964-024-01666-y)

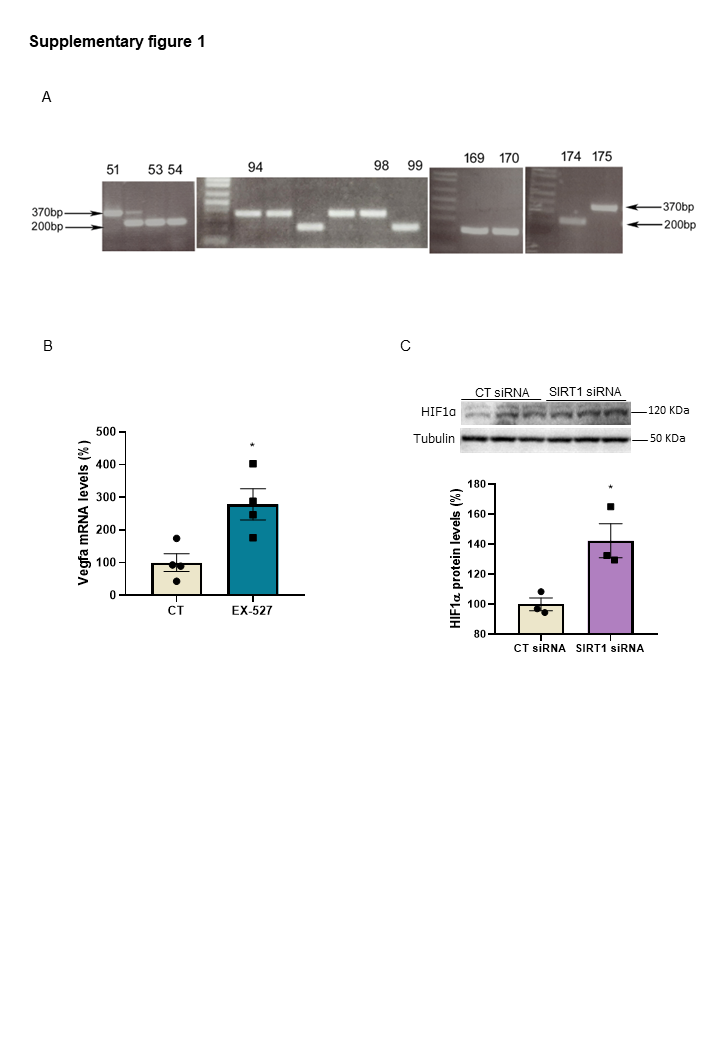

Supplement: Supplementary file 2 — Supplementary Material 2 [file 12964_2024_1666_MOESM2_ESM.tif]

Fig1h

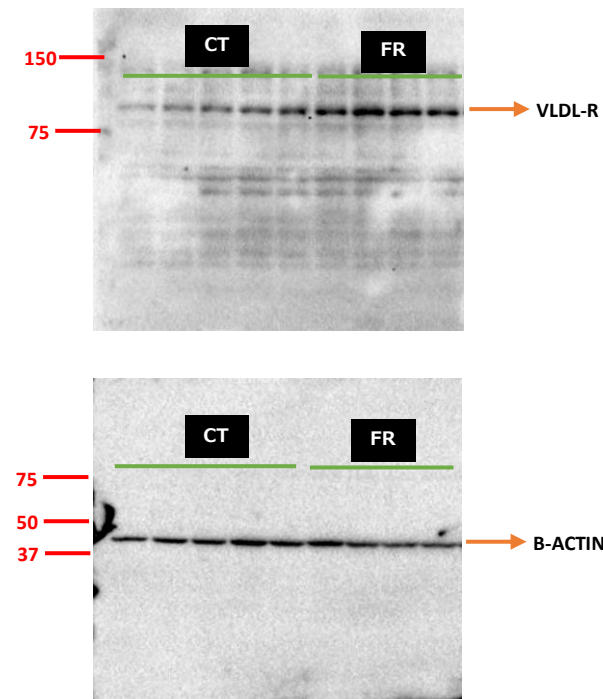

Fig1i

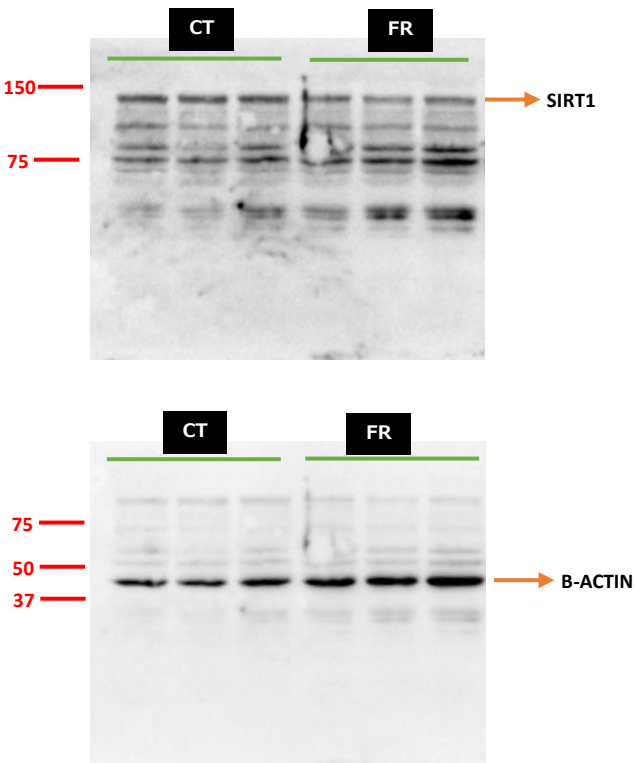

Fig2a

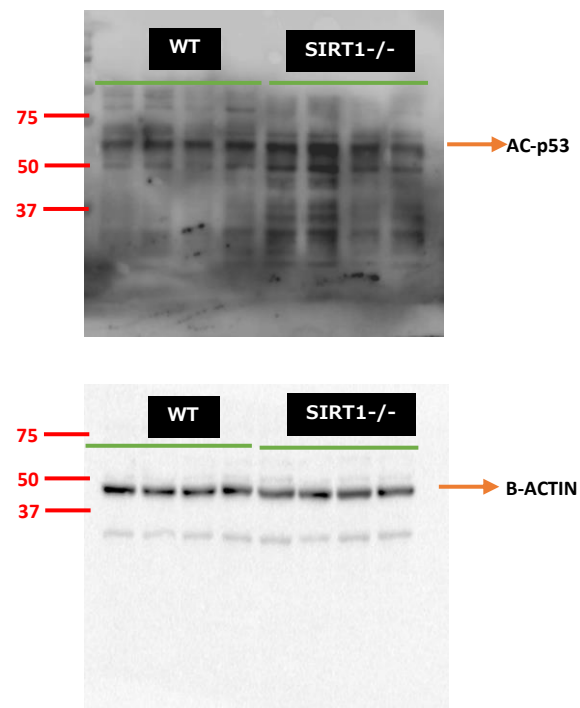

Fig2c

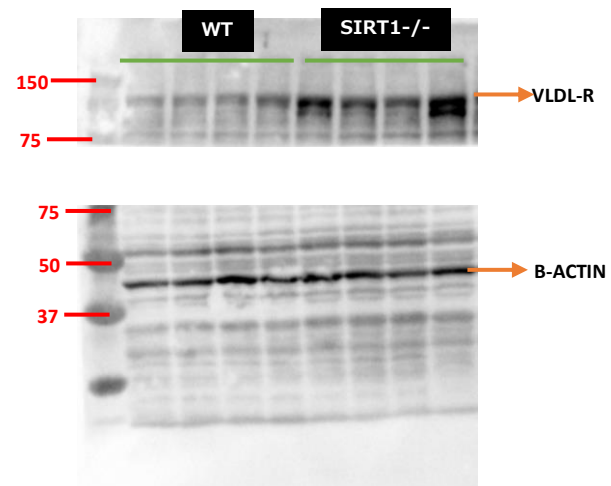

Fig2d

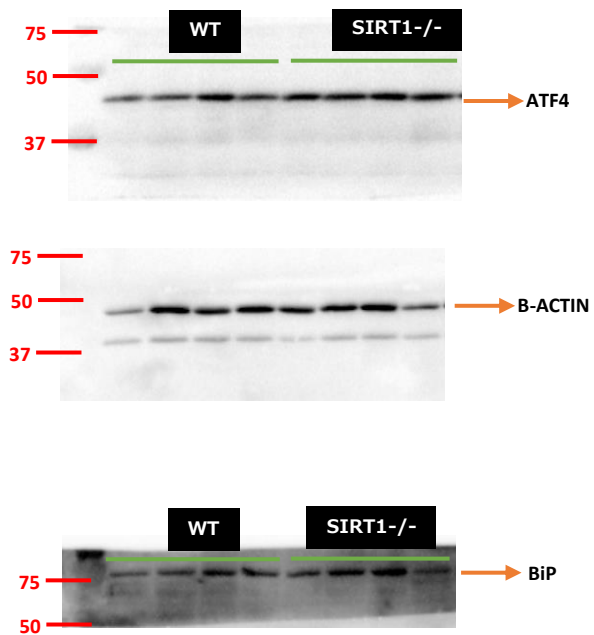

Fig2e

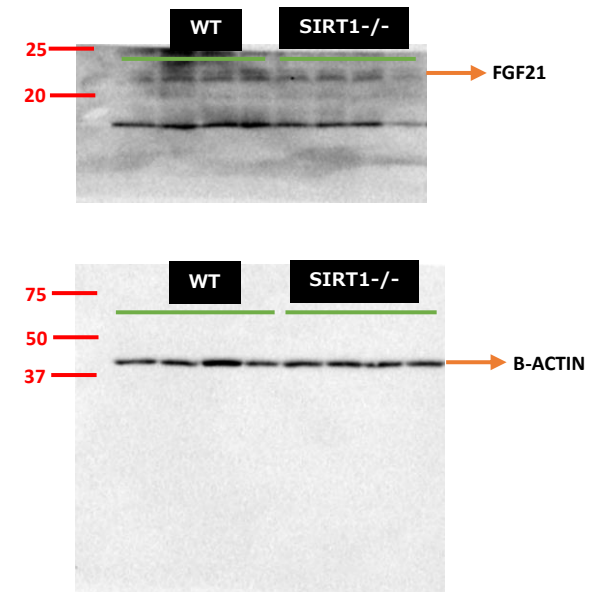

Fig2f

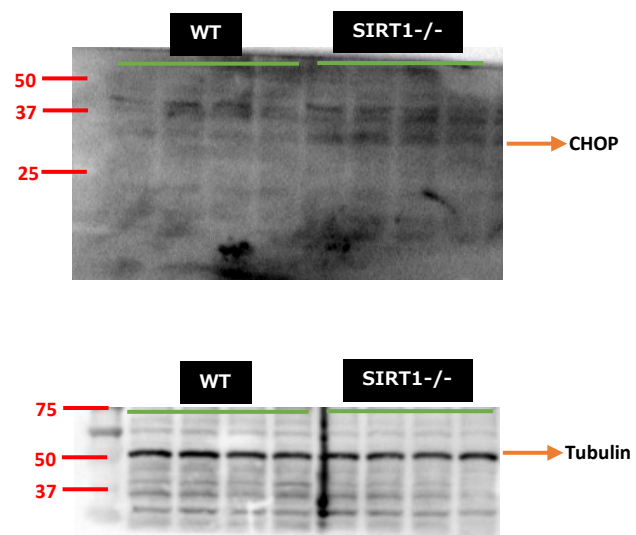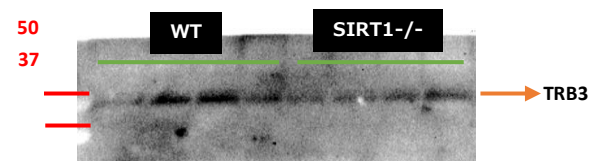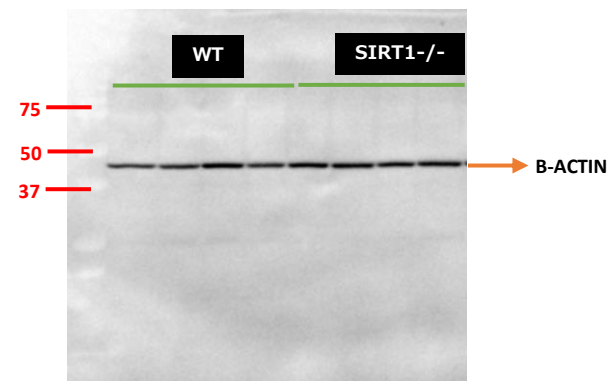

Fig3a

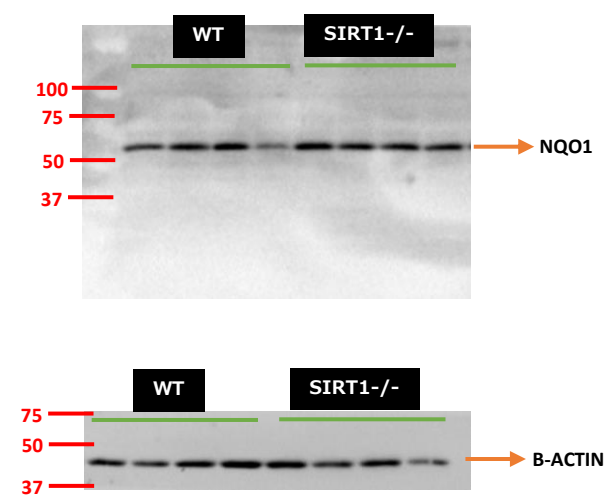

Fig3b

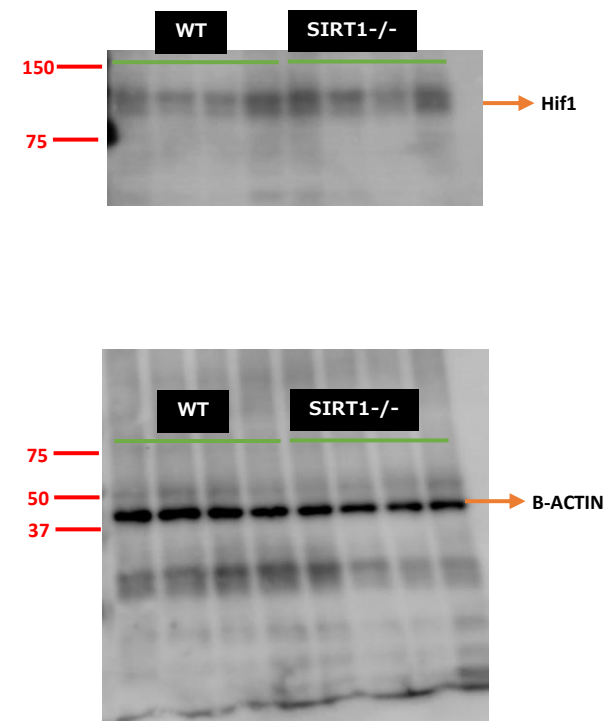

Fig4b

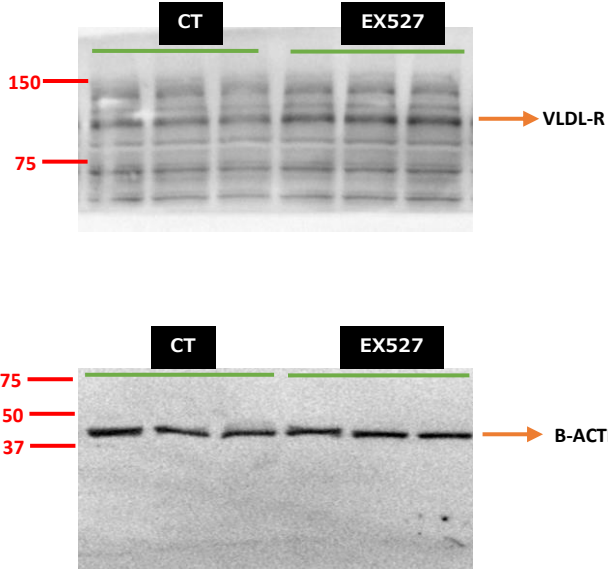

Fig4c

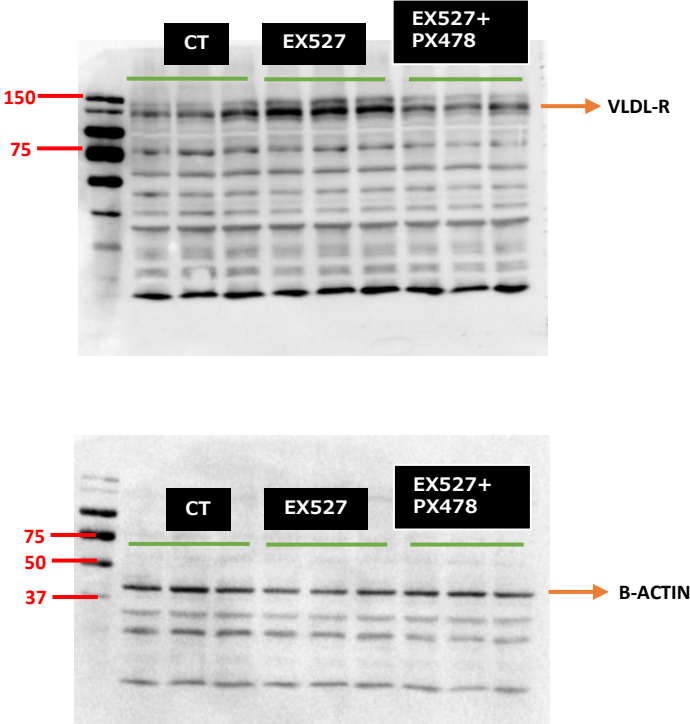

Fig4e

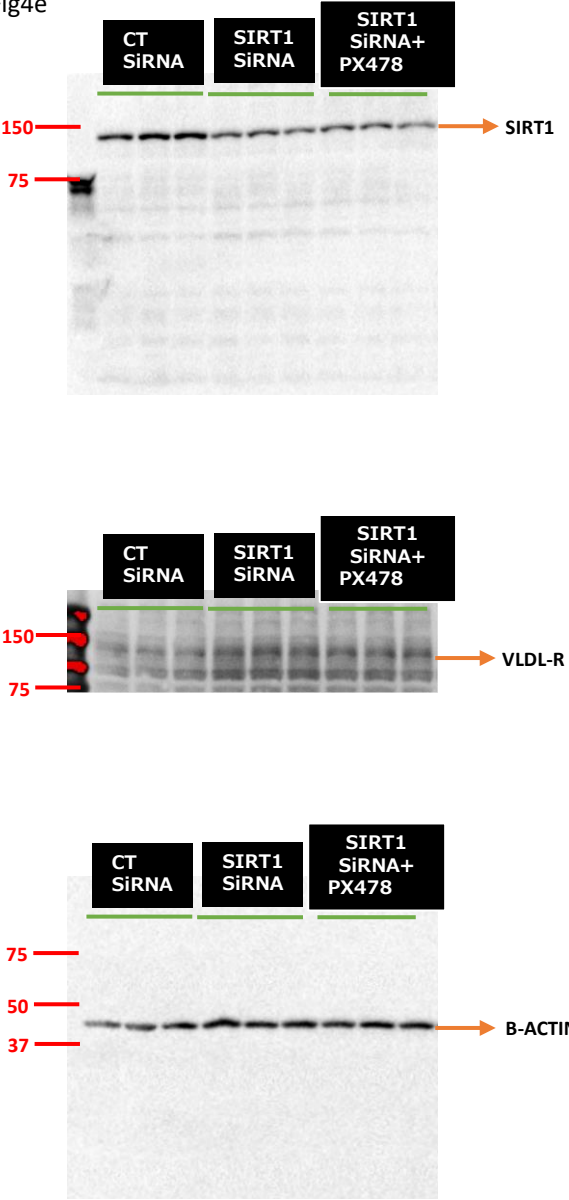

Fig5a

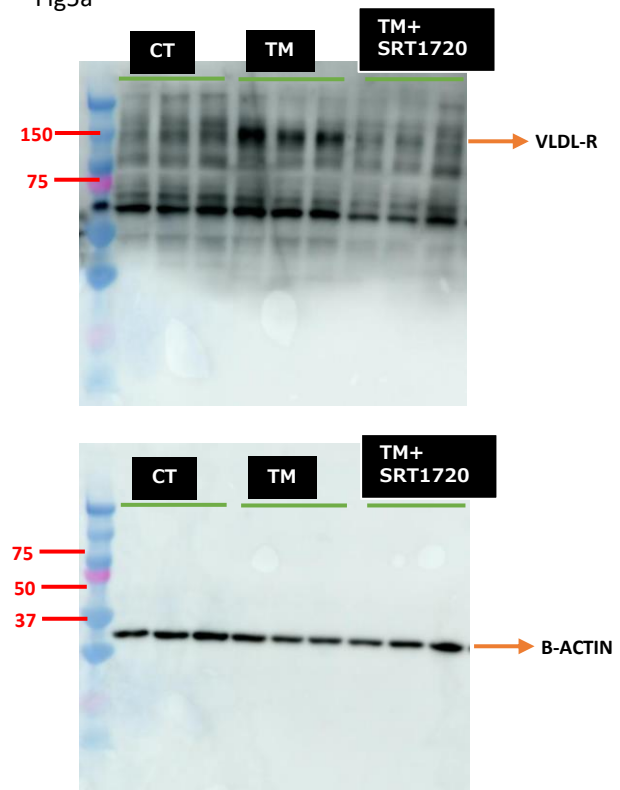

Fig5e

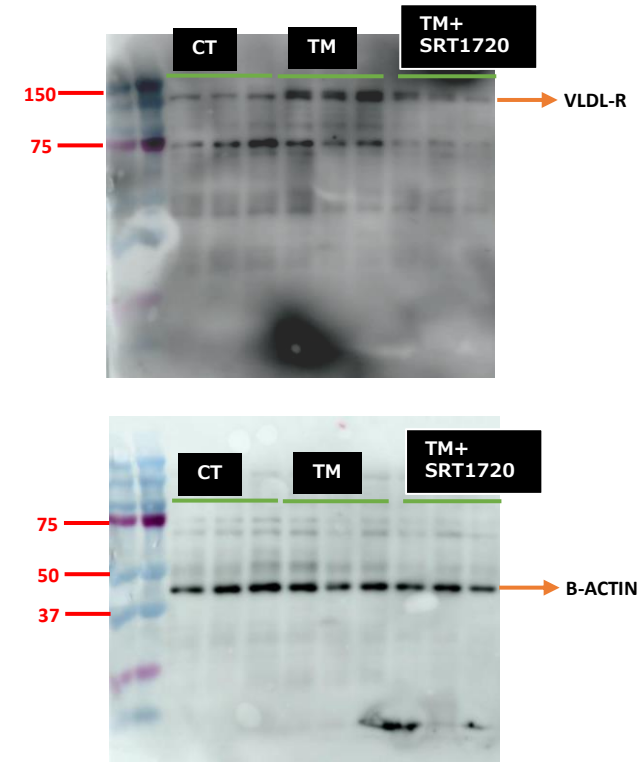

Supplementary Figure 1

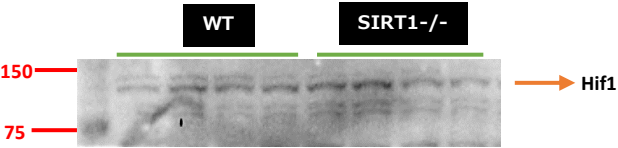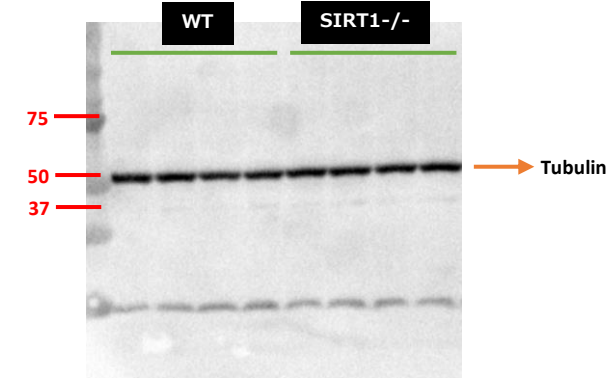

Supplement: Supplementary file 3 — Supplementary Material 3 [file 12964_2024_1666_MOESM3_ESM.pdf]
