# Supplementary material for: SIRT1 regulates hepatic vldlr levels
Source: Cell Commun Signal. 2024 May 28;22:297. doi: 10.1186/s12964-024-01666-y (PMC11134955; doi:10.1186/s12964-024-01666-y)
Supplement: Supplementary file 4 — Supplementary Material 4 [file 12964_2024_1666_MOESM4_ESM.docx]

**Supplementary Fig. 1** (**A**) Genotyping of WT and *Sirt1*^-/-^ mice. Each mouse was genotyped by PCR to differentiate WT and *Sirt1*^-/-^ mice. An image of agarose gels showing the result of representative genotyping is provided. WT: 370 bp. WT: numbers 51, 94, 98, 175. *Sirt1*^-/-^: 200 bp. *Sirt1*^-/-^: numbers 53, 54, 99, 169, 170, 174. (**B**) mRNA levels of *Vegfa* in human Huh-7 cells in the absence (control, CT) or presence of 10 µM EX-527 for 24 h. (**C**) Immunoblot analysis of HIF-1α in human in Huh-7 cells transfected with control siRNA or SIRT1 siRNA for 24 h. Data are presented as the mean ± SEM. Significant differences were established by Student’s t-test. *p < 0.05 *vs.* CT.
